# Linking combined oral contraceptive use to systemic immune marker profiles: the role of cortisol

**DOI:** 10.3389/fendo.2026.1796526

**Published:** 2026-05-11

**Authors:** Johanna Klinger-König, Sandra van der Auwera, Philipp Töpfer, Sabine Ameling, Nele Friedrich, Uwe Völker, Henry Völzke, Johannes Hertel, Hans J. Grabe

**Affiliations:** 1 Department of Psychiatry and Psychotherapy, University Medicine Greifswald, Greifswald, Germany; 2 German Centre for Neurodegenerative Diseases (DZNE), Site Rostock/Greifswald, Greifswald, Germany; 3 Department of Internal Medicine A, University Medicine Greifswald, Greifswald, Germany; 4 Department Functional Genomics, Interfaculty Institute for Genetics and Functional Genomics, University Medicine Greifswald, Greifswald, Germany; 5 German Centre for Cardiovascular Research (DZHK), Partner Site North, Greifswald, Germany; 6 Institute of Clinical Chemistry and Laboratory Medicine, University Medicine Greifswald, Greifswald, Germany; 7 Institute for Community Medicine, University Medicine Greifswald, Greifswald, Germany

**Keywords:** chemokines, cortisol, C-reactive protein, cytokines, growth factors, hormonal contraception, indirect effects, oral contraception

## Abstract

**Introduction:**

Combined oral contraceptive (COC) use has been associated with stress-like, metabolic, and inflammatory alterations. Previous studies demonstrated that metabolic changes were related to cortisol. However, broad immune marker patterns in COC users remain insufficiently characterized, despite evidence of inflammatory involvement. This study investigates COC-related alterations in broad immune marker profiles and whether these are related to cortisol.

**Methods:**

Data from 392 premenopausal women (128 COC users) of the general population-based SHIP-TREND-0 cohort were analyzed using linear models to estimate differences between COC users and non-users. Immune markers included basic inflammation parameters and a broad panel of cytokines, growth factors, and chemokines. Indirect effects through cortisol were tested in structural equation models.

**Results:**

Compared to non-users, COC users had higher cortisol (β=1.24, p<0.001). Higher CRP (β=0.97, p<0.001), and vascular growth factors (i.e., PDGF-AB/BB and VEGF-A; both β=0.27, p<0.05) in COC users were related to cortisol (indirect effects: 0.21 to 0.30; all p<0.05). COC users had fewer monocytes (β=-0.52, p<0.001) and altered chemokine levels (i.e., lower eotaxin and higher MIG levels; β=-0.58 and 0.36, all p<0.001), for which no indirect effects were detected.

**Discussion:**

COC use was associated with broad immune profiling, representing one of the first broad immune marker assessments in COC users. Indirect effects through cortisol suggested both cortisol-dependent and cortisol-independent pathways. These findings provide novel evidence for systemic immunological correlates of COC use and highlight routes of association with endocrine regulation.

## 1 Introduction

Worldwide, hormonal contraception (HC) is the most commonly used contraception method (1). In most Western countries, including Germany, oral contraceptives (OCs) are the most common HC, used by about 150 million women worldwide and approximately 38% of German women of reproductive age (1, 2). OCs either combine estrogen and progestin (combined oral contraceptives; COCs) or use progestin alone.

Like all medications, HC can cause adverse drug reactions (ADRs). About 50% of women discontinue OC use within six to twelve months, with one third justifying their decision by ADRs (3–5). In early studies, similar ADRs were reported for different HC, including bleeding irregularities, mood changes, pain, and nausea (4–8). Accordingly, observational studies regularly comprise women who are continuously taking HC with low or even missing ADRs (“survivor effect”). Nevertheless, altered physiological markers have been described in HC users through partly overlapping pathways: (a) hepatic effects indicated by altered binding globulins and liver markers, (b) metabolic effects associated with stress-like alterations, and (c) altered inflammatory markers.

OCs are metabolized by the liver. More specifically, synthetic estrogens stimulate the hepatic synthesis of sex hormone-binding globulins (SHBGs) and corticosteroid-binding globulins (CBGs) (9–12). CBGs are the primary carrier of cortisol, and their increased availability raises total cortisol levels (13). Contradictory results were found for free cortisol levels, with either unchanged or higher levels (14, 15), the latter also compatible with increased cortisol production (12).

Regulated by the hypothalamic–pituitary–adrenal (HPA) axis, cortisol exerts anti-inflammatory effects during acute stress responses. However, OCs are often used over many years and have been suggested to induce stress-like alterations of HPA-axis regulation, including elevated circulating cortisol (14). Prolonged glucocorticoid exposure may promote glucocorticoid receptor (GR) resistance (i.e., reduced GR sensitivity), thereby attenuating cortisol’s anti-inflammatory signaling and contributing to a positive association between chronic stress and inflammation (15).

In rats, estrogen injections increased the HPA axis activity by attenuating GR-mediated negative feedback through the activation of estrogen receptors in the hypothalamus (16). Moreover, by suppressing the release of luteinizing hormone and follicle-stimulating hormone, COCs reduce endogenous ovarian hormone production. Since endogenous estrogens and progesterone normally exert inhibitory feedback on the HPA axis, their suppression removes this inhibitory brake (17, 18). Consequently, elevated CBG availability and reduced HPA-axis inhibition may interact, linking hepatic steroid-binding changes with altered stress–immune regulation in COC users.

Focusing on the hepatic pathway, progestins variably lower SHBG synthesis, yielding different levels of androgenicity (6). Whereas earlier ethisterone-derived agents were more androgenic, newer progestin generations were designed to minimize these effects (19, 20). Additionally, estrogens have been associated with decreased albumin synthesis, a marker that might reflect hepatic resource allocation, balancing binding-protein synthesis and plasma protein production (21, 22). Moreover, cortisol can bind to albumin, and albumin has been inversely associated with C-reactive protein (CRP) and leukocyte levels (13, 22, 23). These associations provide another link between hepatic and HPA-based stress–immune regulation in COC users.

Higher CRP, leukocyte, and lymphocyte levels were found in COC users compared to non-users (11, 24, 25). However, COC use did not alter cytokine concentrations, such as IL-6 and TNF-α, suggesting predominantly hepatic rather than inflammatory effects (26, 27). Results for transdermal and intrauterine HC are inconsistent, demonstrating either higher or unchanged CRP concentrations (11, 24, 28, 29). Overall, little is known about HC effects on a broad range of inflammatory markers (12, 30). Francis et al. (2016) investigated differences between COC users and HC non-users in cervicovaginal levels of cytokines, chemokines, and growth factors in Tanzanian women (31). They found higher levels of IL-1β, IL-6, IL-8, MIP-1β, and G-CSF in COC users, but did not consider HPA axis co-regulation. Nevertheless, inflammation is coregulated with HPA axis activation and cortisol (32, 33). More precisely, Noushad et al. (2021) have inter alia described cortisol, CRP, IL-6, and triglycerides as potential markers of chronic stress (34).

Hence, cortisol might be a valuable candidate to connect the overlapping pathways and the COC-related changes. Indeed, COC use was associated with increased cortisol levels and other stress-like alterations in the HPA axis (12, 14, 27, 35). Some COC-related lipid changes, including increased triglycerides, could be statistically linked to the higher cortisol levels (14, 36). Additional metabolic alterations have been reported in COC users, such as higher high-density lipoprotein (HDL) levels, lower amino acid concentrations, and higher insulin levels (11, 14, 24, 36). From a clinical perspective, COCs and some of the reported alterations (e.g., higher cortisol, triglyceride, and CRP levels, and lower albumin levels) have been associated with a higher risk of cardiovascular disease (30, 37, 38). In contrast, higher HDL levels and lower platelet-lymphocyte ratios (PLR), also associated with COC use, were associated with decreased cardiovascular risk (39, 40).

However, the underlying biological pathways remain poorly understood. Evidence on connecting parameters, such as cortisol, is only beginning to emerge, and large-scale data on comprehensive immune marker alterations are scarce. The present study uses a set of basic inflammatory markers, cytokines, growth factors, and chemokines to investigate broad inflammatory changes in COC users. Based on a large, general-population sample, it is among the first to provide a comprehensive overview of immune marker alterations associated with COC use. Moreover, we integrate these immune markers with endocrine-metabolic indicators. In more exploratory analyses, we test whether cortisol statistically explains COC-related physiological alterations, which extends existing metabolic findings to a broader immune context.

## 2 Materials and methods

### 2.1 Study cohort

**Figure 1:**
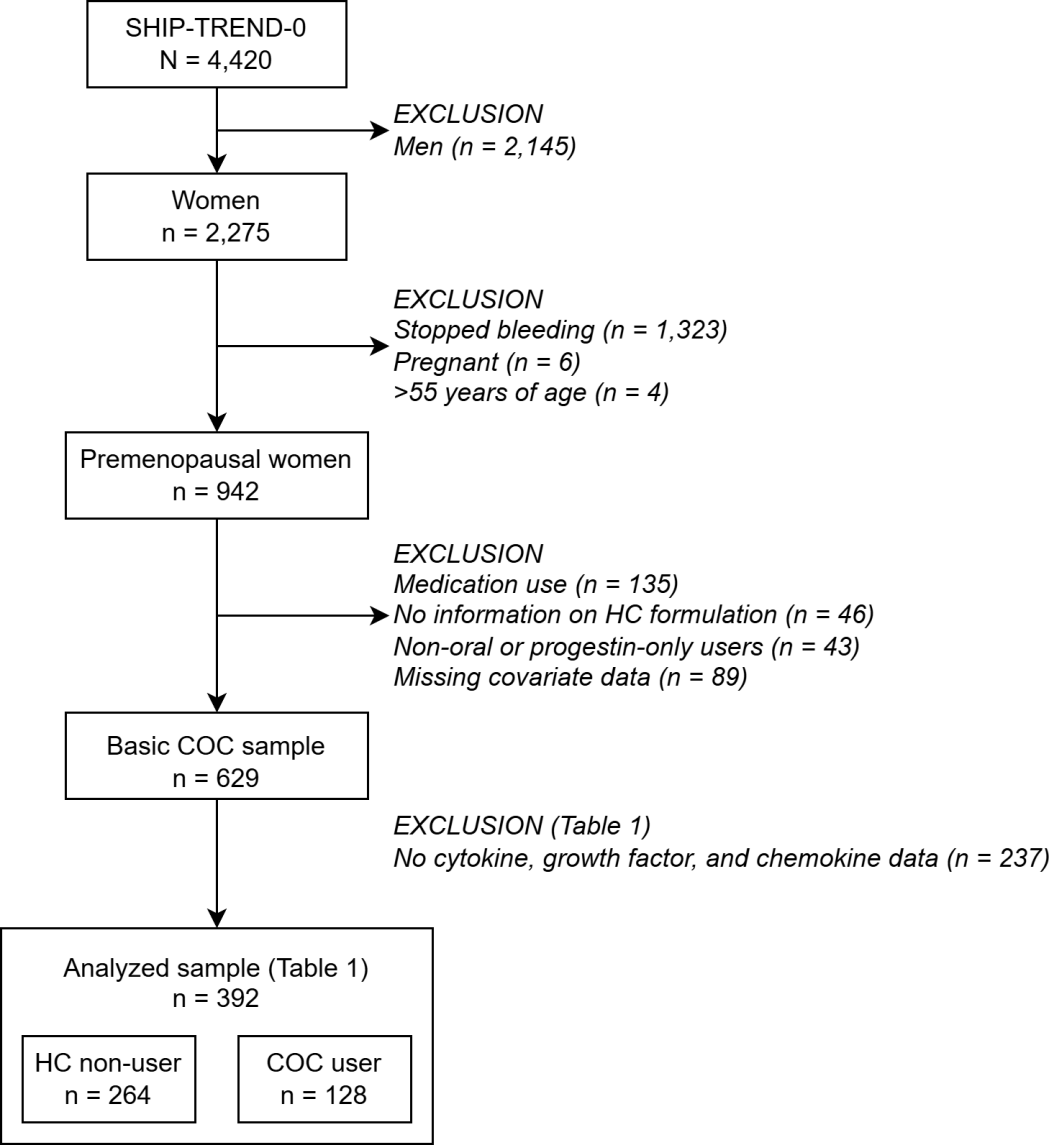
Sample selection and allocation.

Baseline data of one cohort of the Study of Health in Pomerania (SHIP-TREND-0) were used. SHIP is a general population study that covers a broad range of common risk and resilience factors and common characteristics of various (subclinical) diseases. Participants in SHIP-TREND-0 were adults randomly recruited between 2008 and 2012 in Western Pomerania, Northeast Germany, through local registries (N = 4,420). Cytokine, growth factor, and chemokine levels were available for 2,671 of those participants (1,392 women). For the present analyses, we focused on premenopausal women. Men, pregnant and (post-)menopausal women were excluded, as were women who reported the use of potentially interfering medications (see below). The final analytic samples included 264 HC non-users and 128 COC users (**Figure 1**).

### 2.2 Ethics approval

All SHIP-TREND-0 assessments and the analyses presented in this study were conducted according to the Declaration of Helsinki. The study protocol was approved by the Ethics Committee of the University Medicine Greifswald, Germany (approval number: BB 39/08). Written informed consent was obtained from all participants before their participation. All data were processed in a pseudonymized form to ensure participant confidentiality. Only group-level statistics are presented in this study; no individual-level data are reported.

### 2.3 Data collection

#### 2.3.1 Interview data

During a structured, standardized interview, basic sociodemographic data were collected, including sex, age, and education. Education was operationalized as years of formal education, i.e., years of schooling and vocational training (41). Additionally, participants reported their smoking status, allowing differentiation between never, former, and current smokers. Alcohol consumption was determined in grams per day (g/d) over the past 30 days (42). In a women’s questionnaire, women were asked, among others, if they still menstruate, ever and currently use OC, or are currently pregnant. As in previous studies, premenopausal women were required to be still menstruating, be non-pregnant, and be younger than 56 years (**Figure 1**) (14, 36). Additionally, women were asked on the first day of their last withdrawal. The difference between this date and the assessment date was used as the day of the menstrual cycle.

#### 2.3.2 Medication

Participants were asked to report the medications they had used in the past 7 days and to bring their medications or medication sheets. Medications were coded according to the Anatomical Therapeutic Chemical (ATC) classification (ATC-Index, 2007). The present study focused on the effects of COC on the stress and inflammatory systems. Therefore, women reporting the use of the following medications were excluded from the analyses:

- corticosteroids for systemic use (H02).
- acute anti-infectives for systemic use: antibacterials (J01), antimycotics (J02), and antivirals (J05).
- immunosuppressants (L04).
- non-steroidal anti-inflammatories and antirheumatics (M01A).
- sexual hormones other than for contraceptive purposes (G03).

Women without any information on the specific substance, i.e., those identified solely via the women’s questionnaire, were excluded from the analyses (n=48), as were women using another kind of HC except COCs (n=43). COC use was defined as reporting the use of combined HC for systemic use (G03AA or G03AB), or antiandrogens and estrogens (G03HB). These formulations all contain an estrogen–progestin combination with comparable endocrine effects. Although often not primarily prescribed for contraception, G03HB formulations were included because of their similar hormonal composition and expected systemic effects. Former HC users were not distinguished from never users, because 90.0% of current HC non-users reported having used OCs before.

#### 2.3.3 Questionnaire data

The depression module of the Patient Health Questionnaire (PHQ-9) was used to assess depressive symptoms over the past two weeks (43, 44). Nine symptoms are rated on a 4-point scale, and a higher summary score (range: 0-27) indicates more severe depressive symptoms. Additionally, the Toronto-Alexithymia Scale (TAS-20) was used to assess alexithymia, defined as difficulty recognizing or describing emotions (45, 46). Twenty items are rated on a 5-point scale, and higher summary scores (range: 20-125) indicate greater alexithymia.

#### 2.3.4 Anthropometrics

During physical examinations, participants’ height, weight, waist, and hip circumference were manually measured by trained study nurses following a standard operating procedure. For all measurements, participants stood on both feet, without shoes, and wearing light clothing. Height was measured to the nearest 1 cm using a portable stadiometer (Soehnle Industrial Solutions, Backnang, Germany). Waist circumference was measured to the nearest 1 cm using an inelastic tape horizontally midway between the lower rib margin and the iliac crest. The two measurements were integrated into the waist-to-height ratio (WHtR) as an approximation of body fat distribution. In contrast to the commonly used BMI, WHtR is more strongly associated to visceral fat (47). Heart rate, systolic, and diastolic blood pressure were measured three times at the right arm using a digital monitor (HEM-705CP, Omron Corporation, Tokyo, Japan), after an initial rest period of five minutes and with a three-minute rest between measurements. The second and third measurements were averaged for analytical purposes.

#### 2.3.5 Laboratory markers

Blood was drawn from the cubital vein between 7 am and 1.30 pm. Aliquots were stored at -80 °C in the Integrated Research Biobank of the University Medicine Greifswald (48). Participants were asked to fasten before the day of the assessments. The last intake of food and caloric drinks was recorded and used to calculate the fasting time. All laboratory measurements were conducted in accordance with the manufacturer’s instructions.

Serum cortisol was measured using a chemiluminescence immunoassay with low cross-reactivity on the ADVIA Centaur XP System (Siemens Healthcare Diagnostics, Eschborn, Germany). Platelets, leukocytes, and leukocyte types were measured on the XT2000, XE 5000, or SE9000 analyzers from Sysmex (Sysmex Deutschland GmbH, Norderstedt, Germany). Note that proportions of leukocyte types were assessed. Absolute levels were included only for lymphocytes, as these are included in the platelet-lymphocyte ratio (PLR), which was calculated as a potential marker of cardio-metabolic health (25, 49). The Dimension Vista 500 analytical system (Siemens AG, Erlangen, Germany) was used to measure serum concentrations of triglycerides, total cholesterol (Chol), high-density lipoprotein (HDL), low-density lipoprotein (LDL), high-sensitivity C-reactive protein (CRP), albumin, γ-glutamyltransferase (GGT), and bilirubin.

A multiplex assay (HCYTA-60 K-PX48, Merck Millipore, Boston, MA, USA) was used to measure cytokines, growth factors, and chemokines (hereafter referred to as analytes). The results were recorded using the Luminex System (Luminex, Austin, TX, USA), as described elsewhere (50). Overall, 47 analytes were detected. The detection limits for all cytokines have been presented elsewhere (50). For this study, analytes with more than 20% missing concentrations or fewer than 50 valid data points per group (HC non-users/COC users) were excluded (**Supplementary Table 1**). No imputation was performed. Women without any analyte panel data were excluded from the analyses (n=262; **Figure 1**, **Table 1**).

**Table 1:** Sample characteristics of HC non-users and COC users for the analyzed sample and women excluded due to missing cytokine, growth factor, and chemokine data.

Variable	Analyzed		Excluded due to missing analyte data	
	No HC	COC	No HC	COC
N	264	128	151	86
Age [Years]	40.0 [34.0; 45.0]	33.5 [26.8; 41.5]	40.0 [34.5; 44.0]	32.0 [27.0; 37.0]
Smoking				
Never	98 [37.1]	53 [41.4]	46 [30.5]	29 [33.7]
Former	71 [26.9]	30 [23.4]	47 [31.1]	30 [34.9]
Current	95 [36.0]	45 [35.2]	58 [38.4]	27 [31.4]
Formal education	11.0 [11.0; 13.0]	11.0 [11.0; 13.0]	11.0 [11.0; 13.0]	11.0 [11.0; 13.0]
Blood sampling [hh:mm]	09:06 [08:21; 10:46]	09:09 [08:18; 10:45]	09:13 [08:23; 10:56]	09:47 [08:17; 10:05]
Fasting time [hh::mm]	11:29 [03:29; 13:21]	12:41 [10:43; 13:04]	11:14 [03:55; 13:01]	11:38 [02:10; 13:10]
Alcohol consumption [g/d]	2.5 [0.7; 6.0]	2.5 [1.0; 6.5]	2.2 [0.7; 5.9]	3.3 [0.7; 6.6]
WHtR	0.5 [0.4; 0.5]	0.4 [0.4; 0.5]	0.5 [0.4; 0.5]	0.4 [0.4; 0.5]
Menstrual cycle day [d]	14.0 [7.0; 23.0]	14.0 [8.0; 21.0]	13.0 [6.0; 21.0]	14.0 [6.0; 20.8]
COC generation				
not specified		38 [29.7]		21 [24.4]
2^nd^		50 [39.1]		32 [37.2]
3^rd^		25 [19.5]		25 [29.1]
4^th^		15 [11.7]		7 [8.1]
Cortisol [nmol/ml]	265.4 [203.8; 332.2]	512.7 [407.5; 629.1]	252.6 [196.2; 344.6]	464.8 [355.9; 587.9]
CRP [mg/l]	0.9 [0.5; 2.0]	3.0 [1.4; 5.1]	0.9 [0.5; 2.1]	2.4 [1.2; 4.2]
Leukocytes [Gpt/l]	5.9 [5.1; 7.1]	6.1 [5.2; 7.1]	6.0 [5.2; 6.8]	5.9 [5.0; 7.2]
Neutrophils [%]	59.5 [53.3; 64.7]	59.4 [53.6; 65.9]	59.3 [54.4; 62.9]	58.3 [54.9; 64.1]
Monocytes [%]	8.0 [7.0; 9.7]	7.2 [6.0; 8.5]	8.2 [6.9; 9.4]	7.3 [6.1; 8.6]
Eosinophils [%]	2.2 [1.4; 3.2]	1.8 [1.2; 2.9]	2.2 [1.4; 3.0]	1.9 [1.2; 3.2]
Basophils [%]	0.4 [0.3; 0.6]	0.4 [0.3; 0.5]	0.5 [0.3; 0.6]	0.4 [0.2; 0.6]
Lymphocytes [%]	29.1 [24.4; 33.7]	30.4 [25.3; 35.1]	28.8 [25.5; 33.3]	30.7 [26.2; 34.7]
Lymphocytes [Gpt/l]	1.7 [1.4; 2.0]	1.9 [1.6; 2.2]	1.7 [1.4; 2.0]	1.9 [1.5; 2.1]
Platelets [Gpt/l]	241.5 [211.8; 284.0]	251.0 [212.8; 282.0]	257.0 [219.5; 290.5]	248.5 [212.0; 277.0]
PLR	149.7 [119.2; 176.0]	133.1 [107.1; 158.2]	154.0 [123.7; 179.9]	134.7 [112.5; 168.6]
Triglycerides [mmol/l]	0.9 [0.7; 1.4]	1.2 [1.0; 1.7]	1.0 [0.7; 1.4]	1.3 [0.9; 1.6]
HDL [mmol/l]	1.5 [1.3; 1.8]	1.7 [1.4; 2.0]	1.6 [1.3; 1.8]	1.7 [1.5; 1.9]
LDL [mmol/l]	3.1 [2.6; 3.7]	2.9 [2.3; 3.4]	3.1 [2.5; 3.5]	2.8 [2.3; 3.3]
Chol/HDL	3.3 [2.8; 3.9]	3.0 [2.6; 3.6]	3.2 [2.8; 4.0]	2.9 [2.5; 3.5]
Albumin [g/l]	40.0 [38.0; 42.0]	38.0 [36.0; 40.0]	40.0 [38.0; 42.0]	37.0 [36.0; 39.0]
GGT [μkatal/l]	0.4 [0.3; 0.5]	0.4 [0.3; 0.5]	0.4 [0.3; 0.5]	0.4 [0.3; 0.4]
Bilirubin [μmol/l]	6.7 [5.2; 9.2]	6.0 [4.5; 7.6]	6.5 [5.2; 8.8]	5.7 [4.3; 6.7]
Heart rate [beats/min]	75.0 [66.4; 80.5]	74.5 [67.8; 80.5]	73.0 [67.5; 79.5]	75.5 [70.0; 84.5]
systolic BP [mmHg]	112.0 [104.0; 121.5]	112.0 [105.5; 119.5]	111.5 [104.2; 123.0]	111.5 [103.5; 117.5]
PHQ-9	4.0 [2.0; 6.5]	3.0 [1.0; 5.0]	4.0 [2.0; 7.0]	3.0 [2.0; 5.0]
TAS-20	39.0 [34.0; 47.0]	38.0 [33.0; 44.0]	39.0 [35.0; 46.0]	39.0 [32.2; 43.0]

Median [25% percentile; 75% percentile] are presented for continuous and sample size [%] for categorical data. HC, hormonal contraceptives; COC, combined oral contraceptives; WHtR, waist-height ratio; CRP, high-sensitivity C-reactive protein; PLR, platelet-lymphocyte ratio; HDL, high-density lipoprotein; LDL, low-density lipoprotein; Chol/HDL, total cholesterol-HDL ratio; GGT, gamma-glutamyl-transferase; BP, blood pressure; PHQ-9, depression module of the Patient Health Questionnaire; TAS-20, Toronto Alexithymia Scale.

### 2.4 Statistical analyses

Analyses were performed with R 4.5.1. For descriptive purposes, continuous variables are presented as the mean, 25th percentile, and 75th percentile; categorical variables are presented as absolute frequency and percentage.

To determine whether laboratory markers and clinical parameters differed between HC non-users (the reference group) and COC users, weighted general linear models (GLM) with robust standard errors were implemented. Laboratory markers (cortisol, CRP, leukocytes, proportions of leukocyte types, lymphocyte levels, platelets, PLR, triglycerides, HDL, LDL, Chol-HDL ratio [Chol/HDL], albumin, GGT, and bilirubin) and clinical parameters (heart rate, systolic blood pressure, PHQ-9, TAS-20) were used as outcome variables. After visual inspection, extreme outliers were removed, and variables were log-transformed; all outcomes were standardized. All analyses were adjusted for age, day of the menstrual cycle, smoking status (never/former/current), WHtR, and alcohol consumption. Analyses involving laboratory markers were further adjusted for fasting time and time of blood sampling (non-linear). Non-linear associations were modelled using three natural splines. To balance covariate distributions across groups, inverse probability weights for estimating the average treatment effect (ATE) were derived from propensity scores. To account for multiple testing, significance levels were adjusted using the Bonferroni method (p_adj_<0.05). Indirect effects through cortisol were calculated for outcomes that differed between COC users and HC non-users using ATE-weighted structural equation models with robust standard errors (SEM; lavaan package). SEMs were restricted to analytes with nominal significance (p<0.05) in the primary models to reduce dimensionality while retaining potentially informative associations. These analyses were considered exploratory and no correction for multiple testing was applied to the mediation results. Moreover, given the cross-sectional design, indirect effects do not imply temporal order or causal relations. They rather represent model-based decompositions of observed associations as measures of statistical association. All SEMs were adjusted analogous to the GLMs.

To increase focus on immune markers, analyses were repeated using cytokines, growth factors, and chemokines as outcome variables in ATE-weighted GLMs with robust standard errors, adjusted for age, smoking status, WHtR, alcohol consumption, fasting time, time of blood sampling, panel, batch, and storage time. After excluding analytes with high missing rates or low per-group sample size (**Supplementary Table 1**), all analytes were log-transformed and standardized. Robust and extrapolated values were not differentiated. However, sensitivity analyses were conducted solely on valid values. To account for multiple testing, significance levels were adjusted using the Bonferroni method (p_adj_<0.05). Finally, indirect effects through cortisol were calculated for all analytes associated with COC use at an unadjusted level (p<0.05) using SEMs as described above.

To validate the single-analyte findings, a principal component (PC) analysis was calculated (**Supplementary Table 2**). PCs were calculated from ComBat-harmonized cytokine levels, using empirical Bayes methods to adjust the analyte levels for batch, panel, and storage time (51). Missing values were handled using the non-linear iterative partial least squares algorithm. PCs with an eigenvalue greater than 1.0 and an explained variance greater than 5% were used as outcome variables in ATE-weighted GLMs with robust standard errors to estimate differences between HC non-users (the reference group) and COC users, adjusted for age, smoking status (never/former/current), WHtR, alcohol consumption, fasting time, and time of blood sampling (non-linear). To get an impression of the biological correlates, Pearson correlations between the PCs and the covariates, as well as the basic laboratory and clinical parameters, were calculated.

## 3 Results

The final sample comprised 392 women (128 COC users). Descriptively, COC users were younger than HC non-users, smoked less, and had a shorter fasting time (**Table 1**). Power analyses assuming 80% power and a corrected significance threshold of p_adj_<0.05 indicated minimal detectable effect sizes of f²≥0.04 for all contrasts between HC non-users and COC users.

### 3.1 Hepatic, metabolic, and basic inflammatory markers

Main analyses showed differences between HC non-users and COC users for most of the laboratory parameters (**Table 2**). Medium to large effects (f²>0.15; Cohen, 1988) were found for cortisol (β=1.24 [1.08; 1.40], f²=0.60, p_adj_=2.97e-40), CRP (β=0.97 [0.78; 1.16], f²=0.27, p_adj_=2.53e-20), and albumin (β=-0.82 [-1.02; -0.61], f²=0.16, p_adj_=7.41e-13). Additionally, COC users had higher triglyceride (β=0.69 [0.47; 0.90], f²=0.10, p_adj_=4.25e-08) and HDL levels (β=0.37 [0.14; 0.60], f²=0.03, p_adj_=0.044). Hepatic effects were supported by lower bilirubin levels in COC users (β=-0.42 [-0.61; -0.23], f²=0.05, p_adj_=0.001). Moreover, COC users had lower proportions of monocytes (β=-0.52 [-0.74; -0.30], f²=0.06, p_adj_=8.58e-05) and higher absolute lymphocyte levels (β=0.41 [0.20; 0.33], f²=0.04, p_adj_=0.004), potentially supporting immune effects.

**Table 2:** Statistical contrasts for basic laboratory and clinical parameters for COC users compared to HC non-users.

Outcome	β	95%-CI	f²	p-value	p_adj_
Cortisol	1.24	[1.08; 1.40]	0.60	1.35e-41	2.97e-40
CRP	0.97	[0.78; 1.16]	0.27	1.15e-21	2.53e-20
Leukocytes	0.34	[0.11; 0.56]	0.02	0.004	0.088
Neutrophils (%)	0.06	[-0.16; 0.29]	0.00	0.579	1.000
Monocytes (%)	-0.52	[-0.74; -0.30]	0.06	3.90e-06	8.58e-05
Eosinophils (%)	-0.18	[-0.40; 0.04]	0.01	0.106	1.000
Basophils (%)	-0.10	[-0.34; 0.15]	0.00	0.430	1.000
Lymphocytes (%)	0.11	[-0.10; 0.33]	0.00	0.305	1.000
Lymphocytes	0.41	[0.20; 0.62]	0.04	1.71e-04	0.004
Platelets	0.08	[-0.15; 0.30]	0.00	0.505	1.000
PLR	-0.31	[-0.52; -0.11]	0.02	0.003	0.066
Triglycerides	0.69	[0.47; 0.90]	0.10	1.93e-09	4.25e-08
HDL	0.37	[0.14; 0.60]	0.03	0.002	0.044
LDL	-0.20	[-0.39; -0.02]	0.01	0.032	0.704
Chol/HDL	-0.22	[-0.41; -0.02]	0.01	0.029	0.638
Albumin	-0.82	[-1.02; -0.61]	0.16	3.37e-14	7.41e-13
GGT	0.18	[-0.07; 0.43]	0.01	0.154	1.000
Bilirubin	-0.42	[-0.61; -0.23]	0.05	2.37e-05	0.001
Heart rate	-0.11	[-0.34; 0.12]	0.00	0.334	1.000
systolic BP	0.18	[-0.04; 0.40]	0.01	0.108	1.000
PHQ-9	-0.27	[-0.46; -0.07]	0.02	0.007	0.154
TAS-20	-0.17	[-0.38; 0.04]	0.01	0.119	1.000

Outcomes were log-transformed, if necessary, and standardized. Effects are presented for COC users; non-users were used as the reference group for all comparisons. CI, confidence interval; padj, Bonferroni-adjusted p-value; CRP, high-sensitivity C-reactive protein; PLR, platelet-lymphocyte ratio; HDL, high-density lipoprotein; LDL, low-density lipoprotein; Chol/HDL, total cholesterol-HDL ratio; GGT, gamma-glutamyl-transferase; BP, blood pressure; PHQ-9, depression module of the Patient Health Questionnaire; TAS-20, Toronto Alexithymia Scale.

**Figure 2:**
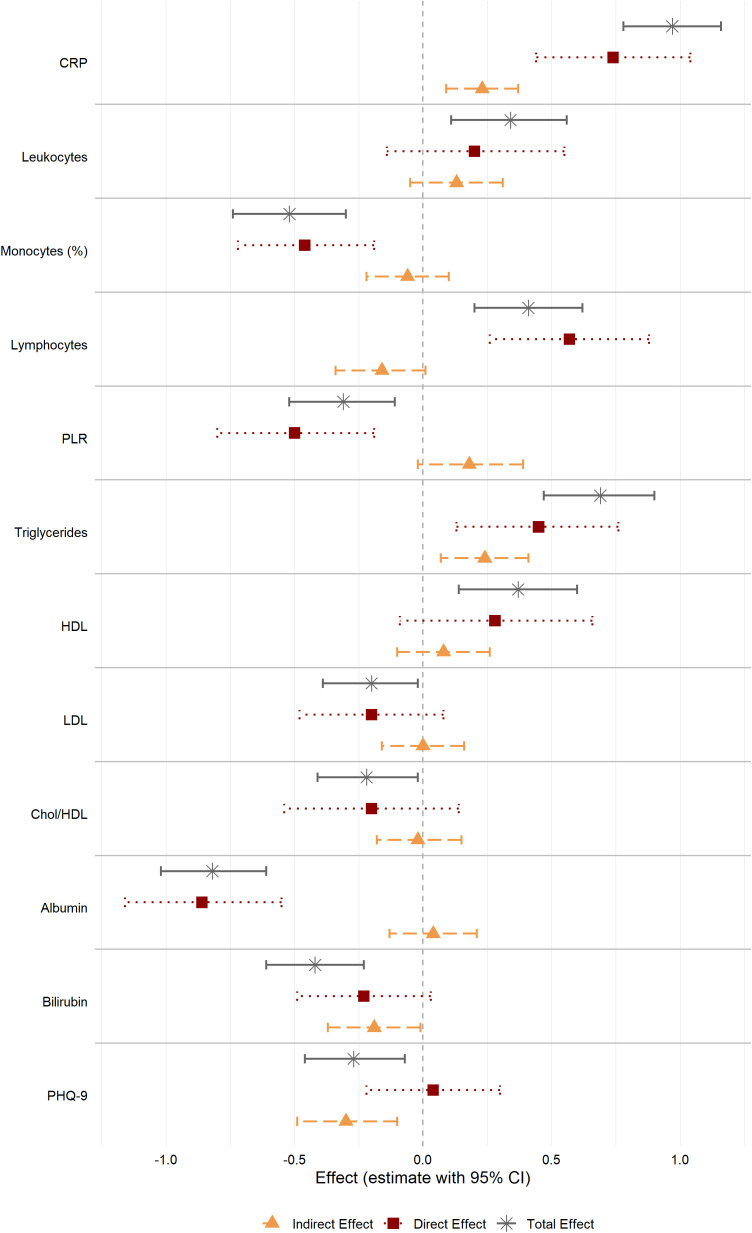
Total, direct and cortisol-related indirect effects for hepatic, metabolic, and basic inflammatory markers in COC users compared to HC non-users. CI, confidence interval.

For some alterations, an evidence for indirect effects through cortisol was uncovered (**Figure 2**; **Supplementary Table 3**). Hence, cortisol partly explained the effects of CRP (indirect effect (IE)=0.23 [0.09; 0.37], p=0.002, 23.7% explained), bilirubin (IE=-0.19 [-0.37; -0.01], p=0.034, 45.8% explained), and triglycerides (IE = 0.24 [0.07; 0.41], p=0.006, 35.0% explained). No evidence for indirect cortisol effects were found in the COC-related alterations of monocyte proportions (IE=-0.06 [-0.22; 0.10], p=0.429), albumin (IE = 0.04 [-0.13; 0.21], p=0.633), and HDL (IE = 0.08 [-0.10; 0.26], p=0.366). For lymphocytes, cortisol was a statistical suppressor (IE=-0.16 [-0.34; -0.01], p=0.067, -40.5% explained), meaning that the direct COC and the indirect, cortisol-related effects went in opposite directions.

### 3.2 Cytokines, growth factors, and chemokines

In single-analyte analyses, COC users had significantly lower levels of eotaxin compared to HC non-users (β=-0.58 [-0.78; -0.38], f²=0.08, p_adj_=1.07e-06) and higher levels of MIG (β=0.36 [0.21; 0.51], f²=0.06, p_adj_=9.82e-05) after correction for multiple testing. Additionally, COC users had lower levels of IP-10 (β=-0.30 [-0.48; -0.11], f²=0.02, p_adj_=175). These two top hits did not contain any extrapolated values (**Supplementary Table 1**). Moreover, differences in IL-18, IP-10, MIP-1β, PDGF-AB/BB, and VEGF-A levels reached a nominal significance level (**Table 3**). All values from IP-10 and PDGF-AB/BB were robust (**Supplementary Table 1**). For IL-18, MIP-1β, and VEGF-A, sensitivity analyses excluding extrapolated values yielded results similar to those with all values (**Table 3**).

**Table 3:** Statistical contrasts for single-analyte analyses and for principal components for COC users compared to HC non-users.

Outcome	All values					Excluding extrapolated values				
	β	95%-CI	f²	p-value	padj	β	95%-CI	f²	p-value	padj
Eotaxin	-0.58	[-0.78; -0.38]	0.08	4.28e-08	1.07e-06					
FLT-3L	0.12	[-0.13; 0.38]	0.00	0.339	1.000	0.25	[0.04; 0.46]	0.02	0.019	0.342
Fractalkine	0.16	[-0.06; 0.39]	0.01	0.158	1.000	0.20	[-0.03; 0.42]	0.01	0.085	1.000
G-CSF	-0.05	[-0.26; 0.17]	0.00	0.681	1.000	0.02	[-0.20; 0.23]	0.00	0.863	1.000
IL-12p40	-0.09	[-0.30; 0.13]	0.00	0.423	1.000	0.02	[-0.18; 0.21]	0.00	0.871	1.000
IL-12p70	0.01	[-0.21; 0.24]	0.00	0.899	1.000	0.29	[0.01; 0.57]	0.02	0.043	0.774
IL-13	0.06	[-0.20; 0.33]	0.00	0.637	1.000	0.19	[-0.06; 0.45]	0.01	0.130	1.000
IL-15	0.05	[-0.19; 0.29]	0.00	0.698	1.000	0.20	[-0.10; 0.50]	0.01	0.190	1.000
IL-18	0.22	[0.08; 0.37]	0.02	0.003	0.075	0.17	[0.05; 0.30]	0.02	0.006	0.108
IL-1RA	-0.01	[-0.25; 0.23]	0.00	0.952	1.000	0.03	[-0.22; 0.27]	0.00	0.826	1.000
IL-27	0.04	[-0.16; 0.24]	0.00	0.703	1.000	0.05	[-0.15; 0.25]	0.00	0.622	1.000
IL-5	0.01	[-0.22; 0.24]	0.00	0.942	1.000	0.07	[-0.15; 0.30]	0.00	0.519	1.000
IL-7	0.07	[-0.16; 0.29]	0.00	0.566	1.000	-0.02	[-0.25; 0.20]	0.00	0.830	1.000
IL-8	0.07	[-0.20; 0.34]	0.00	0.612	1.000	0.17	[-0.08; 0.43]	0.01	0.187	1.000
IP-10	-0.25	[-0.44; -0.07]	0.02	0.007	0.175					
MCP-1	-0.13	[-0.32; 0.06]	0.00	0.174	1.000					
MDC	0.19	[-0.03; 0.40]	0.01	0.094	1.000					
MIG	0.36	[0.21; 0.51]	0.06	3.93e-06	9.82e-05					
MIP-1beta	-0.27	[-0.49; -0.05]	0.01	0.017	0.425	-0.26	[-0.48; -0.04]	0.01	0.018	0.324
PDGF-AA	0.19	[-0.01; 0.39]	0.01	0.060	1.000					
PDGF-AB/BB	0.27	[0.06; 0.47]	0.02	0.012	0.300					
sCD40L	-0.15	[-0.35; 0.05]	0.01	0.153	1.000	-0.14	[-0.33; 0.05]	0.01	0.139	1.000
TNF-alpha	0.14	[-0.08; 0.37]	0.00	0.209	1.000	0.19	[-0.04; 0.41]	0.01	0.108	1.000
TNF-beta	0.18	[-0.07; 0.43]	0.01	0.157	1.000	0.08	[-0.17; 0.32]	0.00	0.551	1.000
VEGF-A	0.27	[0.05; 0.49]	0.02	0.015	0.375	0.29	[0.07; 0.51]	0.02	0.011	0.198
PC 1	0.06	[-0.47; 0.60]	0.00	0.820	1.000					
PC 2	0.11	[-0.21; 0.43]	0.00	0.508	1.000					
PC 3	-0.55	[-0.85; -0.24]	0.03	4.41e-04	0.003					
PC 4	-0.30	[-0.57; -0.03]	0.01	0.033	0.198					
PC 5	-0.26	[-0.48; -0.05]	0.01	0.017	0.102					
PC 6	-0.33	[-0.56; -0.10]	0.02	0.004	0.024					

Analyte levels are log-transformed and standardized. Effects are presented for COC users; non-HC users were used as the reference group. Sensitivity analyses of single-analyte analyses were conducted only on robust values. Principal components are adjusted for technical covariates, namely batch, panel, and storage time. CI, confidence interval.

**Figure 3:**
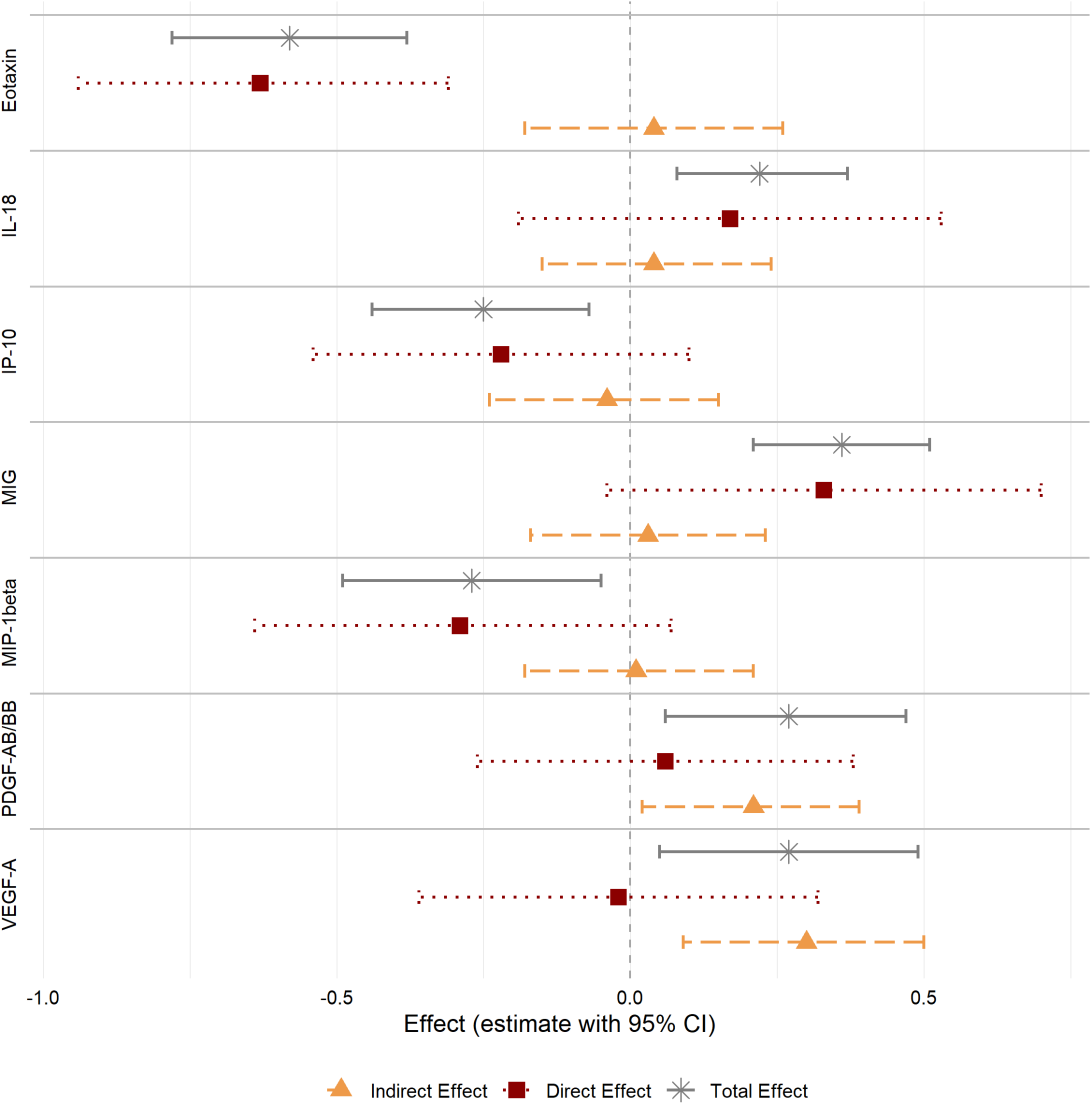
Total, direct and cortisol-related indirect effects for cytokines, growth factors, and chemokines in COC users compared to HC non-users. CI, confidence interval.

Indirect effects for cortisol were found for PDGF-AB/BB (IE=0.21 [0.02; 0.39], p=0.027, 77.1% explained) and VEGF-A (IE=0.30 [0.09; 0.50], p=0.005, 100.0% explained). No indirect effects were observed for the other analytes (**Figure 3**; **Supplementary Table 4**).

The first six PCs were included in PC-based analyses explaining 23% (PC1), 11% (PC2), 10% (PC3), 7% (PC4), and 5% (PC5 and PC6) of the overall variance of the cytokines, chemokines, and growth factors. The PC-based analyses supported the single-analyte findings (**Table 3**; **Supplementary Tables 2**, **5**). Strongest effects were found for PC3 and PC6 (**Table 3**). For these PCs, eotaxin (PC6: λ=0.31), IP-10 (PC6: λ=-0.39), and MIG (PC3: λ=-0.41) had the strongest loadings. Regarding biological and clinical correlations (**Supplementary Table 5**), PC3 showed correlations with leukocytes, lymphocyte proportions, and Tas-20. PC6 correlated with age and CRP. The growth factors, i.e., PDGF-AB/BB and VEGF-A, had the strongest loadings on PC2, which correlated with leukocyte and lymphocyte levels.

## 4 Discussion

This study investigated inflammatory alterations in COC users compared to HC non-users. Replicating previous results, we demonstrated higher cortisol, CRP, and leukocyte levels in COC users (11, 12, 14, 24, 25). In addition to previous studies, we measured a broad panel of cytokines, growth factors, and chemokines. We demonstrated substantial differences in these analyte levels between COC users and HC non-users. However, effect sizes were large for cortisol and medium for CRP, but small for the other variables (52).

As an exploratory strategy, we examined whether higher total cortisol levels could statistically account for the COC-associated alterations. This approach extends the previous metabolic results of Eick et al. (2021) to a broad immune context (36). However, total cortisol levels reflect a broad alteration in the stress regulation system rather than solely biologically active glucocorticoid signaling, and represent only one potential mechanism.

### 4.1 Hepatic, metabolic, and basic inflammatory markers

Increased cortisol levels, reflecting altered HPA axis activity, were a key association in this study and tested as a potential statistical explanation of the observed inflammatory and metabolic profile changes (14, 35, 36). By chronically increasing cortisol exposure, long-term COC use has been suggested to mimic chronic stress (14). Sustained glucocorticoid signaling has been shown to foster GR resistance, weakening anti-inflammatory control and thereby favoring higher inflammation (15).

Estrogens stimulate hepatic CBG synthesis, increasing total cortisol levels in combined HC users, as measured here (9–11). However, other studies have also demonstrated higher free cortisol levels, supporting an additional increase in cortisol production (12, 35). Increased cortisol levels in COC users have already been shown to be related to changes in lipid and metabolite profiles (14, 36). In our data, indirect effects for triglycerides were replicated and extended to CRP, lymphocytes, and growth factors.

Estrogen has been reported to shift the hepatic metabolism toward an increased synthesis of binding proteins, such as SHBG and CBG (9–11). In support of alterations in hepatic metabolism, our data showed lower albumin and bilirubin levels in COC users. Although reduced albumin levels have been associated with estrogen in prior studies, studies investigating albumin and bilirubin in HC users are scarce (21, 22, 53). COC use has been associated with increased plasma volume and capillary permeability, which may contribute to modest reductions in circulating liver markers (54). Moreover, estrogen reduces albumin mRNA expression and increases bilirubin excretion by upregulating bile transporters (12, 21). Although a connection between hepatic metabolism and HPA axis activity in COC users can be inferred (e.g., increased CBG synthesis), cortisol did not explain the effects of albumin in our sample.

In line with previous findings, we could replicate lower PLR (25, 55). The PLR has been described as a prognostic marker for cardiovascular diseases (CVD) (40). However, given the small effect size, the PLR differences are likely of limited clinical relevance in our study. Similarly, effects on other clinical markers, i.e., heart rate, blood pressure, depression, or alexithymia, were limited in our data. COC users often report lower mental and physical symptoms in the general population sample, probably strongly influenced by the survivor effect (14, 56). The lower depressive symptoms and alexithymia levels in our sample might be influenced by this survivor effect. Those effects, however, might be specific to assessments at rest. In response to social stress, COC users reported higher subjective stress and less positive mood compared to HC non-users (57). Interestingly, all associations with basal clinical indicators in our data were related to higher total cortisol levels. In line with this, depression and CVD have been associated with altered HPA axis activity (58–61). Nevertheless, the indirect effects in this study only reflect statistical associations and do not necessarily reflect a biological pathway.

Focusing on basic immune markers, our study found higher CRP and lymphocyte levels in COC users than in HC non-users, replicating previous findings (11, 25). Both CRP and lymphocyte levels were related to cortisol. Both cortisol and CRP have been linked to chronic stress and HPA axis regulation, suggesting a potential co-regulation in this context (34). However, CRP is produced by hepatocytes and thus also reflects hepatic metabolism, as indicated by the inverse association with albumin (23). In our study, total serum cortisol levels were measured; these are influenced by CBG levels, which are also synthesized by the liver. Hence, the association between cortisol and CRP may reflect either shared hepatic regulation or stress-related physiological pathways. In previous studies, COC use was not related to altered cytokine levels, such as TNF-α or IL-6, suggesting that CRP elevation in COC users is not primarily cytokine-driven (26, 27). In line with this, we found only increased IL-18 levels among COC users in our data, with no other cytokine alterations. Overall, we cannot disentangle whether the observed associations reflect hepatic effects, stress-related mechanisms, or a combination of both.

For lymphocytes, the indirect effect through cortisol was negative, indicating a suppressor pathway. COC use increases lymphocyte levels, whereas cortisol exerts an opposing immunosuppressive influence (62). Thus, the total positive association could be a dominant cortisol-independent mechanism, with cortisol partially counteracting this increase. In contrast to the absolute lymphocyte levels, leukocyte levels did not differ between HC non-users and COC users. Regarding the leukocyte compositions, however, COC users had lower proportions of monocytes. Monocytes produce and release various cytokines, such as TNF-α and some interleukins, diminished by estrogen (25). Nevertheless, the lower monocyte proportions were not related to cortisol. Moreover, neither TNF-α nor interleukin levels differed between COC users and HC non-users in our data.

### 4.2 Cytokines, growth factors, and chemokines

The broad investigation of cytokine, growth factor, and chemokine changes in COC users, as conducted in this study, represents a new research approach (12, 30). An earlier study in Tanzanian women reported higher cervicovaginal levels of IL-6, IL-8, MIP-1β, and G-CSF in COC users than in HC non-users (31). These results could not be replicated in our sample, which may support tissue-specific effects. In line, previous results on single serum parameters, such as IL-6 or TNF-α, did not find any associations with COC use (26, 27). Following a social stress test, different associations have been reported between changes in salivary cortisol and changes in salivary levels of the cytokines IL-6 and TNF-α in HC non-users and COC users (57). These results could suggest altered co-regulation of the HPA axis and inflammation in COC users, at least under stress. However, our data focused on basal plasma levels, and it is unclear whether those coupling differences are stress-reaction-specific or also prominent at rest. Nevertheless, our analyses indicate that some analyte levels are altered in COC users. At the single-analyte level, eotaxin and MIG were revealed as the top hits.

Eotaxin (CCL11) is synthesized by lymphocytes, eosinophils, endothelial and epithelial cells, fibroblasts, and macrophages (63). Induced by inflammatory markers, such as various interleukins and TNF-α, it is an important chemokine in allergic reactions, recruiting, activating, and promoting eosinophil survival (63, 64). Additionally, higher levels have been associated with aging, e.g., CVD and lower cognitive performance (50, 65, 66). Eotaxin has been found to cross the blood-brain barrier, and higher eotaxin levels have been associated with reduced neurogenesis and a higher risk of dementia (65, 67). In our study, lower eotaxin levels have been found in COC users than in HC non-users. Cortisol has been reported to lower eotaxin levels (65), which aligns with the higher cortisol levels observed in our COC users. However, we did not observe an indirect effect of cortisol on eotaxin levels in our study.

MIG (CXCL9), produced by monocytes, endothelial cells, fibroblasts, and macrophages, is involved in the development, proliferation, and recruitment of T cells (68). The INF-γ-induced MIG levels were higher in COC users than in HC non-users in our study. In contrast, IP-10 (CXCL10) is synthesized by the same cells as MIG and is also INF-γ-induced, but its level was lower in COC users (68). Moreover, COC users had higher levels of IL-18, a pro-inflammatory cytokine which induces INF-γ and a differentiation of T helper cells 1 (69, 70). MIG and IP-10 exhibit angiostatic effects and have been linked to the inhibition of microvascular endothelial cells, thereby impairing the exchange of substances between blood and surrounding tissue (71). Moreover, both chemokines have been associated with CVD risk, and increased levels have been reported for older participants (72). Similarly, IL-18 was associated with autoimmune diseases, such as rheumatoid arthritis and inflammatory bowel diseases, but also with metabolic syndrome and CVD (70).

In line with lower IP-10 levels, COC users had higher levels of PDGF-AA and PDGF-AB/BB than HC non-users, which are involved in tissue repair, and higher levels of VEGF-A, which is involved in blood vessel genesis (73, 74). These three growth factors are mainly produced by platelets and show pro-angiogenic effects (73, 74). In line, the three analytes had strong negative loadings on PC2, which, in turn, was correlated with lower platelet levels. Although experimental studies often report glucocorticoid-mediated suppression of VEGF and PDGF expression in specific tissues, our data show higher cortisol levels, along with elevated plasma levels of VEGF-A, PDGF-AA, and PDGF-AB/BB, in COC users (75). Still, our models demonstrated a statistical association with cortisol. The complex systemic context of COC use could explain this apparent discrepancy. Within the systemic hormonal environment of COC use, elevated cortisol might reflect a broader endocrine-metabolic shift. Thus, the indirect effects represent a statistical association rather than a necessarily causal stimulatory effect.

Overall, our results replicated previous studies reporting no basal differences in plasma-circulating cytokines (e.g., IL-6 and TNF-α) between HC non-users and COC users. However, we did find differences in other markers that were not previously investigated, such as growth factors (e.g., PDGF-AB/BB and VEGF-A) and chemokines (e.g., eotaxin and MIG). Beyond basal levels, however, findings from a social stress test paradigm suggest that COC use may affect not only the magnitude of endocrine and inflammatory activity but also the pattern of coupling between cortisol and specific immune markers (57). Mengelkoch et al. (2023) found that the associations of stress-induced salivary cortisol changes with IL-6 and TNF-α differed between COC users and naturally cycling women (57). These results may indicate that cortisol–immune relationships are marker-specific and potentially modified by COC use, which could help explain why cortisol was associated with some, but not all, differences in immune markers in our study. However, it remains unclear whether such differential coupling also applies under resting conditions. Additionally, immune markers differ substantially in their biological regulation, including cellular source, temporal response characteristics, and glucocorticoid sensitivity. Consequently, cortisol is unlikely to relate uniformly to all parameters tested here.

### 4.3 Strengths and limitations

The presented results were based on a general population sample of COC users and HC non-users. As women who perceive severe HC ADRs often decide to discontinue usage within the first months, survivor effects in population-based studies are likely (3, 5). Nevertheless, our study still observed metabolic, hepatic, and inflammatory alterations in COC users. If these alterations were accompanied by more severe subjective ADRs, the variations would be even greater among women who decided to discontinue. Moreover, the variability of biological alterations among COC users might differ. Thus, Piltonen et al. (2012) reported large variances in CRP changes in COC users (11).

Our study focused on COC users. The use of other HC types, e.g., progestin-only pills or intrauterine HC, was not investigated due to very low numbers of users. Although Morin-Papunen et al. (2008) found psychological alterations in COC users but not in users of intrauterine HC, Piltonen et al. (2012) demonstrated that the changes in combined HC users were independent of the route of administration (11, 24). These previous findings suggest that the estrogen component is more important for biological alterations than the route of administration. Additionally, COC users were not stratified by the progestin used due to the small sample sizes in those subgroups. Our study extends the existing literature on immune marker changes in COC users by examining a broad panel of immune markers. Hence, our first priority was to produce robust results relying on larger sample sizes. Nevertheless, future studies should replicate our findings in other HC types and differentiate between progestin types.

Furthermore, our analyses are not adjusted for the timing of HC use. The total time spent using OC was assessed during the interview. However, this variable did not differentiate between COC use and progestin-only pills. Similarly, we could not account for differences in prescribing practices. Our sample mainly included middle-aged women and was recruited in 2008. Prescription policies and contraception preferences of women might have changed since then. For example, OC intake is not recommended for women with metabolic challenges, such as hypertension, obesity, diabetes, or dyslipidemia (30, 76, 77).

Although our analyses were based on a large general population sample and used a solid, robust statistical approach, all results are cross-sectional and cannot be interpreted causally. More specifically, the SEM models were used to describe statistically explained proportions and were not intended to represent chronologically ordered biological pathways, nor should they be interpreted as such. Future studies using experimental or longitudinal designs are needed to replicate our findings.

Finally, our analyses included total serum cortisol levels instead of free serum or salivary cortisol levels. Additionally, CBGs were not available for the analyzed sample. Hence, the reported results may not reflect purely biologically active glucocorticoid signaling but rather a potential effect via alterations in the glucocorticoid system.

### 4.4 Conclusion

In conclusion, this study demonstrates broad hepatic, metabolic, and immune alterations in COC users, extending previous work by identifying distinct differences across a wide panel of cytokines, growth factors, and chemokines. Among inflammatory markers, the strongest associations were observed for CRP, lymphocytes, and chemokines. Increased cortisol levels statistically explained some of these alterations, highlighting a potential association between hepatic and stress-immune regulations in COC users. However, these indirect effects represent statistical associations rather than biological mechanisms, underscoring the need for future studies to clarify the physiological pathways underlying COC-related alterations to improve prediction and clinical counseling. Finally, future studies should aim to replicate these findings with free plasma or salivary cortisol levels alongside CBG measurements to deepen the understanding of the biologically active glucocorticoid effects.

## Data Availability

The data underlying this article are not publicly available and cannot be shared by the authors due to SHIP data protection. Researchers may apply for access to the data through the SHIP Transfer Unit (https://transfer.ship-med.uni-greifswald.de/) in accordance with the established application procedures. The present analyses and results are based on the data application SHIP/2025/76/D. Requests to access these datasets should be directed to https://transfer.ship-med.uni-greifswald.de/.
