# Supplementary material for: Linking combined oral contraceptive use to systemic immune marker profiles: the role of cortisol
Source: Front Endocrinol (Lausanne). 2026 May 11;17:1796526. doi: 10.3389/fendo.2026.1796526 (PMC13199129; doi:10.3389/fendo.2026.1796526)
Supplement: Supplementary file 1 [file DataSheet1.docx]

Supplementary Material

Linking combined oral contraceptive use to systematic immune marker profiles: the role of cortisol

J. Klinger-König, S. van der Auwera, P. Töpfer, S. Ameling, N. Friedrich, U. Völker, H. Völzke,
J. Hertel, H. J. Grabe

**Table S1.** Cytokine, growth factor, and chemokine characteristics of HC non-users and COC users.

|  | **All values** | | | | | | **Robust values** | | | | |
| --- | --- | --- | --- | --- | --- | --- | --- | --- | --- | --- | --- |
|  |  | **no HC** | | **COC** | |  |  | **no HC** | | **COC** | |
|  | **% Miss.** | **N** | **Median [25%; 75%]** | **N** | **Median [25%; 75%]** | **analy.** | **% Miss.** | **N** | **Median [25%; 75%]** | **N** | **Median [25%; 75%]** |
| **N max** |  | 264 |  | 128 |  |  |  | 264 |  | 128 |  |
| **EGF** | 28.6 | 194 | 12.4 [5.9; 21.1] | 86 | 11.7 [8.4; 25.3] |  | 33.3 | 149 | 15.4 [9.5; 25.7] | 75 | 14.0 [9.4; 26.7] |
| **Eotaxin** | 0.0 | 264 | 64.7 [51.3; 86.7] | 128 | 45.6 [35.9; 63.5] | x | 0.0 | 264 | 64.7 [51.3; 86.7] | 128 | 45.6 [35.9; 63.5] |
| **FGF-2** | 27.8 | 186 | 46.8 [33.4; 78.5] | 97 | 45.9 [31.2; 64.8] |  | 30.9 | 159 | 53.6 [37.9; 84.6] | 85 | 49.9 [36.7; 70.3] |
| **FLT-3L** | 9.7 | 235 | 6.0 [3.3; 11.1] | 119 | 7.4 [3.5; 12.1] | x | 10.4 | 219 | 6.3 [3.9; 11.4] | 108 | 7.7 [4.2; 12.4] |
| **Fractalkine** | 2.0 | 261 | 95.6 [68.2; 137.0] | 123 | 110.8 [81.5; 152.6] | x | 2.1 | 256 | 97.2 [69.8; 137.6] | 119 | 111.6 [85.8; 154.3] |
| **G-CSF** | 17.6 | 221 | 42.4 [23.6; 69.0] | 102 | 36.4 [22.9; 70.1] | x | 20.7 | 182 | 49.5 [33.9; 74.6] | 83 | 52.2 [28.6; 73.1] |
| **GM-CSF** | 95.2 | 11 | 26.3 [5.3; 63.6] | 8 | 38.4 [8.8; 88.2] |  | 97.9 | 5 | 70.4 [56.8; 94.9] | 3 | 70.8 [65.1; 145.9] |
| **GRO-alpha** | 33.4 | 176 | 6.0 [2.8; 12.2] | 85 | 6.3 [3.6; 11.8] |  | 36.1 | 153 | 7.0 [4.0; 14.2] | 79 | 6.9 [4.0; 12.4] |
| **IFN-alpha2** | 25.3 | 205 | 19.7 [12.2; 31.4] | 88 | 21.2 [13.1; 35.7] |  | 37.2 | 117 | 29.6 [20.3; 40.4] | 50 | 30.6 [23.3; 50.1] |
| **IFN-gamma** | 37.8 | 158 | 7.0 [2.9; 21.7] | 86 | 9.9 [3.6; 31.5] |  | 49.5 | 94 | 12.1 [5.5; 32.3] | 57 | 17.2 [6.0; 41.6] |
| **IL-10** | 37.5 | 169 | 2.6 [1.8; 4.5] | 76 | 2.8 [1.6; 4.2] |  | 56.1 | 79 | 4.7 [3.3; 9.3] | 36 | 4.3 [3.4; 9.7] |
| **IL-12p40** | 2.3 | 258 | 42.0 [26.8; 65.1] | 125 | 40.5 [25.5; 65.0] | x | 2.4 | 245 | 44.1 [28.6; 66.4] | 114 | 44.4 [29.4; 69.0] |
| **IL-12p70** | 15.6 | 225 | 3.5 [2.1; 8.3] | 106 | 3.5 [2.0; 8.5] | x | 25.1 | 129 | 6.9 [4.6; 12.0] | 53 | 8.5 [5.2; 21.1] |
| **IL-13** | 16.3 | 217 | 30.6 [14.3; 70.8] | 111 | 36.9 [16.7; 85.8] | x | 23.6 | 138 | 52.5 [30.0; 105.2] | 69 | 66.3 [36.9; 121.2] |
| **IL-15** | 3.1 | 254 | 3.1 [2.1; 4.3] | 126 | 3.2 [2.2; 5.0] | x | 6.2 | 118 | 4.6 [3.6; 6.8] | 62 | 5.2 [4.2; 6.9] |
| **IL-17A** | 41.3 | 151 | 2.2 [1.1; 4.5] | 79 | 2.1 [1.4; 4.1] |  | 60.4 | 74 | 4.6 [2.9; 8.6] | 32 | 5.4 [3.0; 8.6] |
| **IL-17E/IL-25** | 30.9 | 188 | 123.6 [54.7; 228.4] | 83 | 115.1 [61.8; 229.1] |  | 40.2 | 129 | 182.6 [121.3; 281.9] | 51 | 184.6 [127.4; 465.9] |
| **IL-17F** | 86.2 | 33 | 52.3 [41.5; 90.2] | 21 | 54.0 [37.1; 121.2] |  | 87.6 | 31 | 57.2 [42.0; 90.8] | 17 | 55.5 [48.3; 161.8] |
| **IL-18** | 1.3 | 260 | 52.4 [18.1; 105.7] | 127 | 55.9 [21.3; 144.6] | x | 1.3 | 252 | 55.7 [18.9; 107.5] | 126 | 56.4 [21.6; 144.6] |
| **IL-1alpha** | 45.4 | 139 | 7.0 [4.0; 12.2] | 75 | 7.1 [4.1; 11.1] |  | 85.2 | 19 | 25.8 [18.3; 35.4] | 12 | 35.8 [26.0; 58.7] |
| **IL-1beta** | 27.8 | 193 | 3.6 [1.7; 7.8] | 90 | 3.3 [1.8; 7.0] |  | 38.7 | 115 | 6.2 [3.7; 12.5] | 58 | 4.8 [3.0; 11.2] |
| **IL-1RA** | 7.1 | 247 | 5.1 [3.1; 8.7] | 117 | 5.1 [3.1; 8.1] | x | 7.3 | 243 | 5.2 [3.2; 8.7] | 113 | 5.2 [3.4; 8.2] |
| **IL-2** | 59.2 | 106 | 0.8 [0.5; 1.7] | 54 | 0.8 [0.5; 1.5] |  | 77.1 | 50 | 1.7 [1.2; 3.6] | 19 | 2.0 [1.2; 6.8] |
| **IL-22** | 57.9 | 107 | 74.2 [36.1; 129.5] | 58 | 105.1 [51.2; 151.7] |  | 65.0 | 71 | 100.6 [74.4; 199.5] | 51 | 114.2 [71.9; 156.2] |
| **IL-27** | 0.0 | 264 | 1768.8 [1188.4; 2593.7] | 128 | 1846.8 [1323.0; 2484.6] | x | 0.0 | 263 | 1769.1 [1195.9; 2598.2] | 127 | 1847.4 [1338.4; 2489.2] |
| **IL-3** | 22.2 | 211 | 0.7 [0.5; 1.0] | 94 | 0.7 [0.5; 1.0] |  | 68.5 | 27 | 1.6 [1.5; 2.1] | 13 | 1.9 [1.6; 2.1] |
| **IL-4** | 28.8 | 192 | 1.0 [0.5; 2.0] | 87 | 1.1 [0.5; 2.3] |  | 39.1 | 118 | 1.6 [1.0; 3.0] | 58 | 1.9 [1.1; 3.3] |
| **IL-5** | 0.0 | 264 | 1.7 [1.1; 2.7] | 128 | 1.9 [1.3; 3.0] | x | 0.0 | 252 | 1.8 [1.3; 2.7] | 119 | 2.0 [1.4; 3.1] |
| **IL-6** | 24.2 | 206 | 0.6 [0.3; 1.0] | 91 | 0.7 [0.3; 1.3] |  | 42.2 | 86 | 1.1 [0.9; 1.8] | 44 | 1.3 [0.9; 1.9] |
| **IL-7** | 6.9 | 246 | 1.3 [0.6; 2.3] | 119 | 1.3 [0.8; 2.9] | x | 9.3 | 173 | 1.8 [1.2; 2.8] | 90 | 2.0 [1.2; 3.4] |
| **IL-8** | 17.1 | 219 | 1.6 [1.1; 2.6] | 106 | 1.6 [1.0; 2.6] | x | 18.2 | 207 | 1.7 [1.2; 2.6] | 95 | 1.8 [1.1; 2.7] |
| **IL-9** | 61.2 | 103 | 14.2 [8.2; 35.6] | 49 | 15.4 [8.8; 28.2] |  | 68.2 | 74 | 25.6 [12.9; 40.3] | 38 | 19.2 [11.5; 41.4] |
| **IP-10** | 0.0 | 264 | 168.5 [125.5; 230.8] | 128 | 143.4 [104.4; 200.0] | x | 0.0 | 264 | 168.5 [125.5; 230.8] | 128 | 143.4 [104.4; 200.0] |
| **M-CSF** | 75.0 | 49 | 54.7 [39.3; 65.2] | 49 | 68.1 [46.4; 87.6] |  | 79.0 | 35 | 61.6 [52.7; 79.6] | 43 | 73.4 [54.9; 93.2] |
| **MCP-1** | 0.0 | 264 | 224.8 [180.5; 282.0] | 128 | 206.3 [165.8; 241.9] | x | 0.0 | 264 | 224.8 [180.5; 282.0] | 128 | 206.3 [165.8; 241.9] |
| **MCP-3** | 22.4 | 202 | 26.1 [16.3; 44.9] | 102 | 29.4 [18.0; 50.0] |  | 24.0 | 182 | 28.5 [19.3; 49.3] | 97 | 31.0 [20.0; 51.9] |
| **MDC** | 0.0 | 264 | 507.1 [396.8; 634.2] | 128 | 570.4 [431.1; 702.4] | x | 0.0 | 264 | 507.1 [396.8; 634.2] | 128 | 570.4 [431.1; 702.4] |
| **MIG** | 0.0 | 264 | 858.7 [554.2; 1341.5] | 128 | 1056.2 [646.6; 1743.2] | x | 0.0 | 264 | 858.7 [554.2; 1341.5] | 128 | 1056.2 [646.6; 1743.2] |
| **MIP-1alpha** | 25.3 | 197 | 13.9 [8.9; 24.6] | 96 | 14.2 [10.3; 21.2] |  | 31.6 | 138 | 19.3 [13.0; 30.7] | 76 | 15.8 [13.0; 24.1] |
| **MIP-1beta** | 0.0 | 264 | 18.9 [13.2; 25.7] | 128 | 16.5 [11.7; 21.4] | x | 0.0 | 262 | 18.9 [13.3; 25.7] | 127 | 16.6 [11.9; 21.4] |
| **PDGF-AA** | 0.0 | 264 | 836.0 [442.6; 1793.4] | 128 | 974.1 [516.2; 1633.5] | x | 0.0 | 264 | 836.0 [442.6; 1793.4] | 128 | 974.1 [516.2; 1633.5] |
| **PDGF-AB/BB** | 0.0 | 264 | 7308.0 [4335.3; 12306.1] | 128 | 8108.1 [5479.8; 13366.6] | x | 0.0 | 264 | 7308.0 [4335.3; 12306.1] | 128 | 8108.1 [5479.8; 13366.6] |
| **sCD40L** | 0.8 | 261 | 169.5 [95.4; 338.8] | 128 | 158.5 [86.0; 303.2] | x | 0.8 | 245 | 188.0 [106.9; 354.4] | 118 | 168.3 [96.7; 325.3] |
| **TGF-alpha** | 34.7 | 176 | 2.3 [1.3; 4.9] | 80 | 2.6 [1.7; 6.0] |  | 41.0 | 131 | 3.4 [1.9; 6.4] | 65 | 3.2 [2.0; 7.3] |
| **TNF-alpha** | 3.6 | 254 | 20.0 [12.9; 29.3] | 124 | 23.0 [14.0; 31.7] | x | 3.8 | 241 | 20.6 [13.7; 30.2] | 115 | 23.5 [15.6; 33.4] |
| **TNF-beta** | 9.2 | 240 | 4.7 [2.4; 8.9] | 116 | 5.8 [2.8; 13.6] | x | 10.9 | 195 | 5.8 [3.4; 11.2] | 100 | 7.3 [3.5; 17.1] |
| **VEGF-A** | 2.6 | 257 | 15.4 [7.0; 33.8] | 125 | 18.7 [9.0; 44.1] | x | 2.7 | 237 | 17.0 [8.3; 37.3] | 117 | 23.7 [11.4; 46.7] |
| *Analytes are expressed in pg/ml. Analytes with a missing rate of more than 20% or fewer than 50 women in either group were excluded from the principal analytes analyses. Miss.=Missing; anly.=analyzed; HC=hormonal contraceptives; COC=combined oral contraceptives; CI=confidence interval* | | | | | | | | | | | |

**Table S2.** Principal components for the analyzed cytokines, growth factors, and chemokines.

|  | **PC1** | **PC2** | **PC3** | **PC4** | **PC5** | **PC6** | **PC7** | **PC8** | **PC9** | **PC10** |
| --- | --- | --- | --- | --- | --- | --- | --- | --- | --- | --- |
| **Eotaxin** | 0.11 | 0.09 | 0.27 | 0.17 | 0.39 | 0.31 | 0.33 | 0.06 | 0.11 | 0.00 |
| **FLT-3L** | 0.15 | -0.08 | -0.29 | 0.21 | -0.02 | 0.27 | 0.21 | -0.07 | -0.13 | 0.37 |
| **Fractalkine** | 0.29 | -0.14 | -0.02 | -0.35 | 0.13 | -0.00 | 0.10 | -0.04 | 0.00 | -0.03 |
| **G-CSF** | 0.14 | -0.01 | 0.27 | 0.15 | -0.13 | -0.07 | -0.01 | 0.16 | -0.18 | -0.23 |
| **IL-12p40** | 0.19 | -0.21 | 0.03 | 0.10 | -0.01 | -0.09 | -0.43 | 0.18 | 0.62 | 0.33 |
| **IL-12p70** | 0.22 | -0.06 | 0.14 | -0.16 | 0.03 | 0.09 | -0.04 | 0.02 | -0.03 | 0.07 |
| **IL-13** | 0.25 | -0.11 | -0.09 | -0.34 | 0.14 | 0.04 | 0.03 | -0.03 | -0.10 | -0.02 |
| **IL-15** | 0.20 | -0.23 | -0.25 | 0.15 | -0.21 | 0.29 | -0.05 | 0.02 | -0.03 | 0.01 |
| **IL-18** | 0.09 | -0.09 | 0.36 | 0.10 | -0.18 | -0.16 | 0.04 | 0.00 | -0.26 | 0.25 |
| **IL-1RA** | 0.22 | -0.18 | 0.03 | 0.23 | -0.31 | -0.01 | -0.01 | 0.17 | 0.03 | -0.32 |
| **IL-27** | 0.00 | -0.13 | -0.34 | 0.02 | 0.40 | -0.06 | -0.07 | 0.66 | -0.10 | -0.30 |
| **IL-5** | 0.24 | -0.18 | -0.21 | 0.14 | -0.28 | 0.15 | 0.11 | -0.13 | -0.18 | -0.21 |
| **IL-7** | 0.19 | 0.20 | -0.02 | -0.08 | -0.15 | -0.12 | -0.37 | 0.27 | -0.28 | 0.32 |
| **IL-8** | 0.22 | 0.04 | 0.06 | 0.11 | -0.09 | 0.26 | -0.10 | 0.07 | -0.06 | 0.13 |
| **IP-10** | 0.15 | -0.11 | 0.06 | 0.37 | 0.31 | -0.39 | -0.19 | -0.40 | -0.13 | -0.17 |
| **MCP-1** | 0.16 | 0.07 | 0.25 | 0.27 | 0.34 | 0.24 | 0.11 | 0.10 | -0.01 | 0.20 |
| **MDC** | 0.13 | -0.02 | -0.03 | 0.09 | -0.16 | -0.53 | 0.60 | 0.30 | 0.14 | 0.19 |
| **MIG** | 0.06 | -0.09 | -0.41 | 0.22 | 0.27 | -0.24 | -0.07 | -0.21 | -0.14 | 0.26 |
| **MIP-1beta** | 0.24 | 0.01 | 0.25 | 0.08 | 0.09 | -0.01 | -0.25 | 0.03 | -0.05 | -0.19 |
| **PDGF-AA** | 0.20 | 0.48 | -0.17 | -0.01 | 0.05 | -0.05 | -0.04 | -0.01 | -0.04 | -0.09 |
| **PDGF-AB/BB** | 0.21 | 0.49 | -0.16 | -0.03 | -0.02 | -0.04 | -0.00 | 0.00 | 0.01 | -0.03 |
| **sCD40L** | 0.26 | 0.17 | -0.13 | 0.07 | -0.09 | 0.01 | 0.08 | -0.22 | 0.53 | -0.22 |
| **TNF-alpha** | 0.29 | -0.20 | 0.12 | -0.16 | 0.09 | -0.18 | 0.08 | -0.08 | -0.01 | -0.00 |
| **TNF-beta** | 0.23 | -0.14 | 0.03 | -0.45 | 0.08 | -0.01 | 0.03 | -0.12 | -0.03 | 0.02 |
| **VEGF-A** | 0.23 | 0.36 | 0.04 | 0.01 | -0.09 | -0.08 | 0.01 | -0.02 | -0.07 | 0.02 |
| **R²** | 0.23 | 0.11 | 0.10 | 0.07 | 0.05 | 0.05 | 0.04 | 0.03 | 0.03 | 0.03 |
| **Cum. R²** | 0.23 | 0.34 | 0.44 | 0.51 | 0.56 | 0.61 | 0.65 | 0.69 | 0.72 | 0.75 |
| **Eigenvalue** | 5.17 | 2.44 | 2.18 | 1.61 | 1.19 | 1.14 | 0.93 | 0.77 | 0.68 | 0.62 |
| *Principal components are based on log-transformed and standardized analyte levels and are adjusted for technical covariates, namely batch, panel, and storage time. PC=principal component; Cum.=Cumulative* | | | | | | | | | | |

**Table S3.** Total, direct, and cortisol-related indirect effects for basic laboratory parameters in COC users compared to HC non-users.

|  | **Direct Effect** | | | **Indirect Effect** | | | **Total Effect** | | | **Explained** |
| --- | --- | --- | --- | --- | --- | --- | --- | --- | --- | --- |
|  | **β** | **95%-CI** | **p-value** | **β** | **95%-CI** | **p-value** | **β** | **95%-CI** | **p-value** | **%** |
| **CRP** | 0.74 | [0.44; 1.04] | 1.30e-06 | 0.23 | [0.09; 0.37] | 0.002 | 0.97 | [0.75; 1.20] | <2.20e-16 | 23.72 |
| **Leukocytes** | 0.20 | [-0.14; 0.55] | 0.253 | 0.13 | [-0.05; 0.31] | 0.150 | 0.34 | [0.07; 0.60] | 0.013 | 39.75 |
| **Monocytes (%)** | -0.46 | [-0.72; -0.19] | 0.001 | -0.06 | [-0.22; 0.10] | 0.429 | -0.52 | [-0.75; -0.29] | 1.08e-05 | 12.44 |
| **Lymphocytes** | 0.57 | [0.26; 0.88] | 3.26e-04 | -0.16 | [-0.34; 0.01] | 0.067 | 0.41 | [0.18; 0.63] | 4.75e-04 | -40.47 |
| **PLR** | -0.50 | [-0.80; -0.19] | 0.002 | 0.18 | [-0.02; 0.39] | 0.084 | -0.31 | [-0.53; -0.10] | 0.004 | -58.21 |
| **Triglycerides** | 0.45 | [0.13; 0.76] | 0.005 | 0.24 | [0.07; 0.41] | 0.006 | 0.69 | [0.44; 0.93] | 4.31e-08 | 34.97 |
| **HDL** | 0.28 | [-0.09; 0.66] | 0.133 | 0.08 | [-0.10; 0.26] | 0.366 | 0.37 | [0.11; 0.63] | 0-006 | 22.70 |
| **LDL** | -0.20 | [-0.48; 0.08] | 0.163 | 0.00 | [-0.16; 0.16] | 0.994 | -0.20 | [-0.40; -0.01] | 0.046 | 0.30 |
| **Chol/HDL** | -0.20 | [-0.54; 0.14] | 0.250 | -0.02 | [-0.18; 0.15] | 0.841 | -0.22 | [-0.45; 0.02] | 0.067 | 7.85 |
| **Albumin** | -0.86 | [-1.16; -0.55] | 2.78e-08 | 0.04 | [-0.13; 0.21] | 0.633 | -0.82 | [-1.04; -0.59] | 7.33e-13 | -5.09 |
| **Bilirubin** | -0.23 | [-0.49; 0.03] | 0.083 | -0.19 | [-0.37; -0.01] | 0.034 | -0.42 | [-0.62; -0.22] | 5.10e-05 | 45.82 |
| **PHQ-9** | 0.04 | [-0.22; 0.30] | 0.763 | -0.30 | [-0.49; -0.10] | 0.003 | -0.26 | [-0.46; -0.06] | 0.011 | 100.00 |
| *Outcomes and cortisol were log-transformed, if necessary, and standardized. Effects are presented for combined oral contraceptive users; non-users were used as the reference group. CI=confidence interval; CRP=high-sensitivity C-reactive protein; PLR=platelet-lymphocyte ratio; HDL=high-density lipoprotein; LDL=low-density lipoprotein; Chol/HDL=total cholesterol - high-density lipoprotein ratio; BP=blood pressure* | | | | | | | | | | |

**Table S4.** Total, direct, and cortisol-related indirect effects for principal components of cytokines, growth factors, and chemokines, as well as single-analyte analyses for COC users compared to HC non-users.

|  | **Direct Effect** | | | **Indirect Effect** | | | **Total Effect** | | | **Explained** | |  |
| --- | --- | --- | --- | --- | --- | --- | --- | --- | --- | --- | --- | --- |
|  | **β** | **95%-CI** | **p-value** | **β** | **95%-CI** | **p-value** | **β** | **95%-CI** | **p-value** | **%** |  |  |
| **Eotaxin** | -0.63 | [-0.94; -0.31] | 8.48e-05 | 0.04 | [-0.18; 0.26] | 0.709 | -0.58 | [-0.81; -0.36] | 5.57e-07 | -7.14 |  |  |
| **IL-18** | 0.17 | [-0.19; 0.53] | 0.344 | 0.04 | [-0.15; 0.24] | 0.658 | 0.22 | [0.01; 0.43] | 0.047 | 20.59 |  |  |
| **IP-10** | -0.22 | [-0.54; 0.10] | 0.185 | -0.04 | [-0.24; 0.15] | 0.649 | -0.26 | [-0.47; -0.06] | 0.013 | 17.17 |  |  |
| **MIG** | 0.33 | [-0.04; 0.70] | 0.080 | 0.03 | [-0.17; 0.23] | 0.770 | 0.36 | [0.14; 0.59] | 0.002 | 8.16 |  |  |
| **MIP-1beta** | -0.29 | [-0.64; 0.07] | 0.116 | 0.01 | [-0.18; 0.21] | 0.880 | -0.27 | [-0.54; -0.01] | 0.045 | -5.49 |  |  |
| **PDGF-AB/BB** | 0.06 | [-0.26; 0.38] | 0.705 | 0.21 | [0.02; 0.39] | 0.027 | 0.27 | [0.04; 0.50] | 0.022 | 77.14 |  |  |
| **VEGF-A** | -0.02 | [-0.36; 0.32] | 0.915 | 0.30 | [0.09; 0.50] | 0.005 | 0.28 | [0.03; 0.52] | 0.026 | 100.00 |  |  |
| *Outcomes and cortisol were log-transformed, if necessary, and standardized. Effects are presented for combined oral contraceptive users; non-users were used as the reference group. CI=confidence interval; CRP=high-sensitivity C-reactive protein; PLR=platelet-lymphocyte ratio; HDL=high-density lipoprotein; Chol/HDL=total cholesterol - high-density lipoprotein ratio; BP=blood pressure* | | | | | | | | | | | | |

**Table S5.** Biological and clinical correlations with the first six principal components of the cytokines, chemokines and growth factors.

|  | **PC1** | **p-value** | **p_adj_** | **PC2** | **p-value** | **p_adj_** | **PC3** | **p-value** | **p_adj_** | **PC4** | **p-value** | **p_adj_** | **PC5** | **p-value** | **p_adj_** | **PC6** | **p-value** | **p_adj_** |
| --- | --- | --- | --- | --- | --- | --- | --- | --- | --- | --- | --- | --- | --- | --- | --- | --- | --- | --- |
| **Age** | -0.06 | 0.207 | 1.000 | 0.14 | 0.004 | 0.100 | -0.09 | 0.066 | 1.000 | 0.24 | 2.48e-06 | 6.20e-05 | 0.20 | 7.29e-05 | 0.002 | 0.16 | 0.001 | 0.025 |
| **WHtR** | 0.03 | 0.549 | 1.000 | -0.02 | 0.736 | 1.000 | 0.01 | 0.921 | 1.000 | 0.20 | 6.25e-05 | 0.002 | -0.15 | 0.003 | 0.075 | -0.14 | 0.004 | 0.100 |
| **Alcohol Consump.** | 0.02 | 0.724 | 1.000 | 0.06 | 0.219 | 1.000 | -0.07 | 0.163 | 1.000 | 0.03 | 0.514 | 1.000 | 0.02 | 0.676 | 1.000 | 0.10 | 0.052 | 1.000 |
| **Cortisol** | 0.02 | 0.708 | 1.000 | 0.10 | 0.058 | 1.000 | -0.19 | 1.81e-04 | 0.005 | -0.06 | 0.202 | 1.000 | -0.11 | 0.038 | 0.950 | -0.07 | 0.178 | 1.000 |
| **CRP** | 0.11 | 0.030 | 0.750 | 0.02 | 0.703 | 1.000 | -0.10 | 0.054 | 1.000 | 0.06 | 0.267 | 1.000 | -0.17 | 0.001 | 0.025 | -0.29 | 5.96e-09 | 1.49e-07 |
| **Leukocytes** | 0.01 | 0.788 | 1.000 | 0.17 | 0.001 | 0.025 | 0.13 | 0.009 | 0.225 | -0.03 | 0.558 | 1.000 | -0.2 | 7.17e-05 | 0.002 | -0.13 | 0.010 | 0.250 |
| **Neutrophils (%)** | -0.02 | 0.647 | 1.000 | 0.12 | 0.016 | 0.400 | -0.01 | 0.777 | 1.000 | 0.02 | 0.678 | 1.000 | 0.00 | 0.964 | 1.000 | -0.13 | 0.009 | 0.225 |
| **Monocytes (%)** | 0.05 | 0.311 | 1.000 | -0.04 | 0.426 | 1.000 | -0.03 | 0.588 | 1.000 | 0.11 | 0.023 | 0.575 | 0.18 | 2.54e-04 | 0.006 | 0.06 | 0.253 | 1.000 |
| **Eosinophils (%)** | 0.09 | 0.081 | 1.000 | -0.08 | 0.105 | 1.000 | 0.04 | 0.386 | 1.000 | 0.13 | 0.012 | 0.300 | -0.17 | 0.001 | 0.025 | -0.01 | 0.774 | 1.000 |
| **Basophils (%)** | -0.01 | 0.820 | 1.000 | 0.00 | 0.940 | 1.000 | 0.03 | 0.595 | 1.000 | 0.05 | 0.346 | 1.000 | 0.09 | 0.086 | 1.000 | -0.05 | 0.310 | 1.000 |
| **Platelets (%)** | -0.01 | 0.803 | 1.000 | -0.10 | 0.055 | 1.000 | 0.01 | 0.864 | 1.000 | -0.09 | 0.083 | 1.000 | -0.01 | 0.909 | 1.000 | 0.13 | 0.008 | 0.200 |
| **Lymphocytes (%)** | 0.00 | 0.928 | 1.000 | 0.10 | 0.047 | 1.000 | 0.15 | 0.003 | 0.075 | -0.11 | 0.033 | 0.825 | -0.20 | 8.19e-05 | 0.002 | 0.00 | 0.982 | 1.000 |
| **Lymphocytes** | 0.06 | 0.255 | 1.000 | 0.18 | 4.66e-04 | 0.012 | 0.04 | 0.434 | 1.000 | -0.04 | 0.442 | 1.000 | -0.10 | 0.047 | 1.000 | 0.02 | 0.691 | 1.000 |
| **PLR** | 0.06 | 0.221 | 1.000 | 0.05 | 0.351 | 1.000 | -0.09 | 0.083 | 1.000 | 0.07 | 0.182 | 1.000 | 0.11 | 0.027 | 0.675 | 0.02 | 0.746 | 1.000 |
| **Triglycerides** | 0.05 | 0.284 | 1.000 | 0.03 | 0.583 | 1.000 | -0.04 | 0.486 | 1.000 | 0.11 | 0.033 | 0.825 | -0.25 | 3.27e-07 | 8.18e-06 | -0.10 | 0.050 | 1.000 |
| **HDL** | -0.02 | 0.715 | 1.000 | 0.04 | 0.401 | 1.000 | -0.10 | 0.048 | 1.000 | -0.13 | 0.009 | 0.225 | 0.15 | 0.002 | 0.050 | 0.07 | 0.155 | 1.000 |
| **LDL** | -0.06 | 0.224 | 1.000 | 0.13 | 0.008 | 0.200 | 0.00 | 0.951 | 1.000 | 0.14 | 0.004 | 0.100 | -0.07 | 0.148 | 1.000 | -0.06 | 0.262 | 1.000 |
| **Chol/HDL** | -0.03 | 0.498 | 1.000 | 0.06 | 0.276 | 1.000 | 0.04 | 0.449 | 1.000 | 0.17 | 0.001 | 0.025 | -0.19 | 1.28e-04 | 0.003 | -0.09 | 0.081 | 1.000 |
| **Albumin** | 0.05 | 0.366 | 1.000 | 0.02 | 0.671 | 1.000 | 0.06 | 0.258 | 1.000 | -0.05 | 0.322 | 1.000 | 0.08 | 0.123 | 1.000 | 0.06 | 0.221 | 1.000 |
| **GGT** | -0.08 | 0.124 | 1.000 | 0.00 | 0.997 | 1.000 | 0.00 | 0.954 | 1.000 | 0.15 | 0.002 | 0.050 | -0.01 | 0.871 | 1.000 | -0.09 | 0.069 | 1.000 |
| **Bilirubin** | -0.08 | 0.111 | 1.000 | -0.06 | 0.219 | 1.000 | -0.06 | 0.281 | 1.000 | 0.01 | 0.835 | 1.000 | 0.13 | 0.009 | 0.225 | 0.03 | 0.573 | 1.000 |
| **Heart rate** | 0.09 | 0.062 | 1.000 | 0.00 | 0.959 | 1.000 | 0.00 | 0.956 | 1.000 | 0.00 | 0.951 | 1.000 | -0.07 | 0.168 | 1.000 | -0.10 | 0.041 | 1.000 |
| **systolic BP** | 0.03 | 0.604 | 1.000 | 0.03 | 0.588 | 1.000 | -0.03 | 0.539 | 1.000 | 0.10 | 0.051 | 1.000 | -0.07 | 0.156 | 1.000 | -0.04 | 0.379 | 1.000 |
| **PHQ-9** | -0.03 | 0.502 | 1.000 | 0.00 | 0.956 | 1.000 | -0.09 | 0.078 | 1.000 | 0.00 | 0.985 | 1.000 | -0.02 | 0.761 | 1.000 | 0.07 | 0.175 | 1.000 |
| **TAS-20** | 0.02 | 0.752 | 1.000 | 0.00 | 0.939 | 1.000 | -0.16 | 0.001 | 0.025 | -0.09 | 0.086 | 1.000 | -0.05 | 0.335 | 1.000 | 0.03 | 0.504 | 1.000 |
| *PC=Principal Component; p_adj_=Bonferroni-adjusted p-value; WHtR=waist-height ratio; Alcohol Consump.=Alcohol Consumption; CRP=high-sensitivity C-reactive protein; PLR=platelet-lymphocyte ratio; HDL=high-density lipoprotein; LDL=low-density lipoprotein; Chol/HDL=total cholesterol - high-density lipoprotein ratio; GGT=gamma-glutamyl-transferase; BP=blood pressure; PHQ-9=Depression module of the Patient Health Questionnaire; TAS-20=Toronto Alexithymia Scale* | | | | | | | | | | | | | | | | | | |
